# Targeting microRNAs as a Therapeutic Strategy to Reduce Oxidative Stress in Diabetes

**DOI:** 10.3390/ijms20246358

**Published:** 2019-12-17

**Authors:** Giuseppina Emanuela Grieco, Noemi Brusco, Giada Licata, Laura Nigi, Caterina Formichi, Francesco Dotta, Guido Sebastiani

**Affiliations:** 1 Diabetes Unit, Department of Medicine, Surgery and Neurosciences, University of Siena, V.le Bracci, 16, 53100 Siena, Italy; giusy.grieco.90@gmail.com (G.E.G.); noemibrusco91@gmail.com (N.B.); giadalicata.92@gmail.com (G.L.); launigi@gmail.com (L.N.); catefo@libero.it (C.F.); sebastianiguido@gmail.com (G.S.); 2 Fondazione Umberto Di Mario ONLUS c/o Toscana Life Sciences, 53100 Siena, Italy; 3 UO Diabetologia, Azienda Ospedaliera Universitaria Senese, 53100 Siena, Italy

**Keywords:** diabetes, oxidative stress, miRNAs, drug delivery, aptamers, nanoparticles

## Abstract

Diabetes mellitus is a group of heterogeneous metabolic disorders characterized by chronic hyperglycaemia as a consequence of pancreatic β cell loss and/or dysfunction, also caused by oxidative stress. The molecular mechanisms involved inβ cell dysfunction and in response to oxidative stress are also regulated by microRNAs (miRNAs). miRNAs are a class of negative gene regulators, which modulate pathologic mechanisms occurring in diabetes and its complications. Although several pharmacological therapies specifically targeting miRNAs have already been developed and brought to the clinic, most previous miRNA-based drug delivery methods were unable to target a specific miRNA in a single cell type or tissue, leading to important off-target effects. In order to overcome these issues, aptamers and nanoparticles have been described as non-cytotoxic vehicles for miRNA-based drug delivery. These approaches could represent an innovative way to specifically target and modulate miRNAs involved in oxidative stress in diabetes and its complications. Therefore, the aims of this review are: (i) to report the role of miRNAs involved in oxidative stress in diabetes as promising therapeutic targets; (ii) to shed light onto the new delivery strategies developed to modulate the expression of miRNAs in diseases.

## 1. Introduction

Diabetes mellitus is a chronic metabolic disorder characterized by hyperglycaemia and insulin secretion impairment caused by β cell dysfunction or death. The most common forms of diabetes mellitus are Type 1 Diabetes (T1D), which represents 5–10% of diabetes cases and is caused by immune mediated destruction of β cells, and Type 2 Diabetes (T2D), which represents 90–95% of diabetes cases and is caused by insulin resistance and β cell dysfunction [1]. However, both T1D and T2Doften lead to several types of chronic complications (e.g., retinopathy, neuropathy, nephropathy, and cardiovascular diseases) which affect different organs [2]. 

In such a context, a pivotal role is played by oxidative stress which represents the outcome of an imbalance between the production and neutralization of Reactive Oxygen Species (ROS) and nitrogen species (NS) and which leads to a reduced antioxidant capacity of the cell. In diabetes, oxidative stress is mainly caused by an excessive increase of glucose, Free Fatty Acids (FFA), and/or inflammatory mediators which, in turn, cause mitochondrial dysfunction and/or Endoplasmic Reticulum (ER) stress with consequent insulin resistance and β cell dysfunction. In vascular endothelial cells these stress stimuli lead to the accumulation of Nitric Oxide (NO), increased Advanced Glycation-End products (AGE), activation of polyol-sorbitol pathway, Diacylglycerol (DAG), and Protein Kinase C (PKC) mediators, as well as to an increase of cytokines and prostanoids concentration.

These molecular pathways are activated in distinct cellular conditions and in specific cell types. The factors involved in the development and exacerbation of oxidative stress are tightly regulated by several genetic and epigenetic factors, and, among these, the involvement of microRNAs (miRNAs) has been largely described and demonstrated by several studies. MiRNAs are small endogenous non coding RNAs of 19–24 nucleotides which negatively regulate gene expression. The direct binding of the miRNA seed sequence on the 3′Untranslated Region (3′ UTR) of target gene leads to degradation of mRNA or repression of protein translation [3].

Multiple studies have shown that miRNAs are pivotal regulators of different aspects of glucose homeostasis, such as peripheral insulin signaling [4], β cells function [5], and phenotype maintenance [6,7].

Of note, studies have demonstrated that several miRNAs regulate the expression and function of genes involved in pathways leading to oxidative stress in β cells and in tissues involved in diabetic chronic complications [8,9]. Therefore, it could be important to develop therapeutic strategies based on the modulation of the expression of these miRNAs, in order to inhibit or stimulate their activity and to partially relieve cellular dysfunctions caused by oxidative stress.

Different strategies (such as nanoparticles or aptamers) have been adopted to deliver miRNA-based drugs. Thus, the aim of this review is to shed light on the real putative clinical application of miRNA-based drugs which could potentially target oxidative stress molecular mechanisms involved in diabetes and its complications.

## 2. Oxidative Stress in Diabetes

Under the conditions of elevated metabolism or enhanced mitochondrial activity, many tissues/cells could be the target of Reactive Oxygen Species (ROS) insults. The ROS-mediated damage of tissues/cells has been demonstrated to be involved indifferent conditions including inflammation, age-related degeneration, cancer, and diabetes) [10,11,12]. In particular, chronic exposure to elevated concentration of glucose, Fatty Acids (FAs), and/or inflammatory mediators can damage different types of cells through several mechanisms [13,14]:

(i) Increased glucose flux and other sugars through the polyol pathway.
(ii) Increased intracellular formation of Advanced Glycation End-products (AGEs).
(iii) Increased expression of receptors for AGEs and its activating ligands.
(iv) Activation of Protein Kinase C (PKC) isoforms and downstream pathways.
(v) Over-activity of the hexosamine pathway [15].

Several studies indicate that these mechanisms are activated by a single upstream event: mitochondrial overproduction of ROS [11]. Additionally, compared to many other cell types, β cells may be at high risk of oxidative damage, showing: (i) excessive levels of mitochondrial ROS generation; (ii) additional ROS generation through elevated β cell Nicotinamide Adenine Dinucleotide Phosphate (NADPH) oxidase activity; (iii) failure of antioxidant defense [16]. In β cells, the generation of ROS could be caused by several conditions including hyperglycaemia, hyperlipidaemia, hypoxia, and Endoplasmic Reticulum (ER) stress [17]. During chronic hyperglycaemia, β cells are exposed to high glucose concentrations for an extended period of time. In such a context, glycolytic processes can become saturated; as a consequence, an excess of glucose is shunted towards alternative ROS-forming pathways such as glycosylation, glucose autoxidation, and the glucosamine pathway [18], which leads to the accumulation of ROS and induction of oxidative stress. In addition to hyperglycaemia, exposure to excessive lipids levels has also been shown to activate cellular stress responses leading to oxidative stress [19]. The mechanism by which Free Fatty Acids (FFAs) promotes ROS generation in mitochondria could be explained by the activation of NADPH oxidase as shown by Morgan et al. [20]. Another mechanism which contributes to lipid-induced oxidative stress in βcells is the modulation of the respiratory chain by FFAs. Indeed, βcells exposed to FFAs exhibit increased ROS production through mitochondrial respiratory complex-I involvement [21]. Pancreatic βcells are also prone to the stress caused by low oxygen level which leads to ROS production and other signs of oxidative stress [22]. Hypoxia or low oxygen tension can lead to increased ROS generation, mostly through the involvement of complexes-I and III of the mitochondrial electron transport chain [22,23].

ER stress is also closely related to oxidative stress. In fact, ER stress occurs when the level of misfolded proteins exceeds ER adaptive capabilities, leading to the activation of signaling events which causes the reduction of insulin transcription and translation [24]. In the ER, ROS are generated as a byproduct of protein folding events; therefore, the increased accumulation of dysregulated formation or breakage of disulfide bonds results in an excessive amount of ROS which causes oxidative stress [25]. Additionally, ER stress activates CCAAT/Enhancer Binding Protein (C/EBP) homologous protein (CHOP), demonstrated to be involved in the induction of oxidative stress [26].

Although many pathways may lead to abnormal ROS generation, the main signal leading to oxidative stress in β cells during diabetes is represented by chronic hyperglycaemia. In physiologic conditions, glucose is transported across the plasma membrane [via the specific membrane transporters Glucose Transporter 1 (GLUT1) or Glucose Transporter 2 (GLUT2)] and is rapidly phosphorylated by a specific glucokinase enzyme with high Km for glucose. The combination of transport and phosphorylation determines the exacerbation of glycolytic events in β cells, which result in a rapid rise in the production of reducing equivalents, enhanced activity of shuttle mechanisms responsible for transferring electrons to the mitochondrial matrix, and enhanced Tricarboxylic Acid Cycle (TCA) activity, thus leading to increased Adenosine Triphosphate (ATP) production in mitochondria and to increased ATP to Adenosine Diphosphate (ADP) ratio. This event induces the closure of the ATP-sensitive K^+^ channels (K-ATP), decreasing the hyperpolarizing outward K^+^ flux, resulting in depolarization of the plasma membrane, influx of extracellular Ca^2+^, a rapid increase in intracellular Ca^2+^, and activation of kinases, which finally mediate exocytosis of insulin [27]. 

However, in contrast to other mammalian cell types, increased glucose concentration in β cells stimulates a rapid and proportional rise of glycolytic flux followed by a robust stimulation in the production of reducing equivalents, due to channeling of glucose carbon into the TCA cycle which can lead to increased ROS production. The elevation of intracellular Ca^2+^, induced by increased influx through voltage-gated Ca^2+^ channels, is the primary driver of the Glucose-Stimulated Insulin Secretion (GSIS) mechanism. However, further increase of intracellular Ca^2+^ can stimulate mitochondrial generation of ROS, while Ca^2+^—via PKC activation, may enhance NADPH oxidase-dependent generation of ROS, thus inducing oxidative stress and/or apoptosis [20,28,29]. Of note, the low levels of β cell antioxidant defenses [free radical detoxifying and redox-regulating enzymes, such as Glutathione Peroxidase (GPx), catalase, and thioredoxin] render the oxidative stress particularly detrimental for islet function and survival [16,30]. In addition, the presence of Superoxide Dismutase (SOD), causes the abnormal accumulation of H_2_O_2_ and other ROS [31] which can damage the cells at multiple levels [16]. 

**Figure 1:**
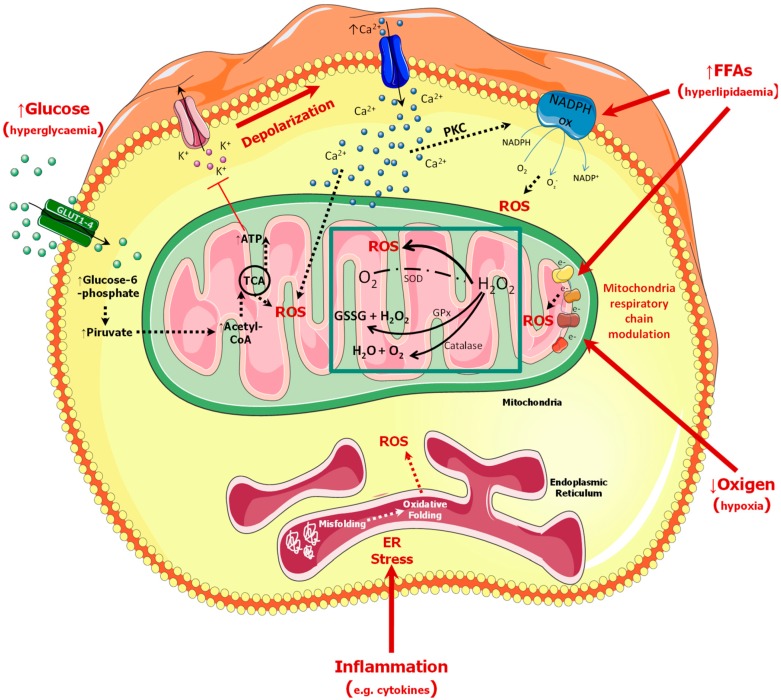
Graphical representation of oxidative stress mechanisms in β cells. The generation of Reactive Oxygen Species (ROS) could be caused by several conditions including hyperglycaemia, hyperlipidaemia, hypoxia, and Endoplasmic Reticulum (ER) stress (due to inflammation). Increased glucose concentration in β cells stimulates a rapid and proportional rise of glycolytic flux followed by a robust stimulation in the production of reducing equivalents, due to the channeling of glucose carbon into the Tricarboxylic Acid Cycle (TCA) cycle, which can lead to an enhancement of ROS production. However, further increases in intracellular Ca^2+^ can stimulate mitochondrial generation of ROS while Ca^2+^ via Protein Kinase C (PKC) activation, may enhance Nicotinamide Adenine Dinucleotide Phosphate (NADPH) oxidase-dependent generation of ROS and, thus, induce oxidative stress and/or apoptosis. The mechanism by which Free Fatty Acids (FFAs) promotes ROS generation in mitochondria could be explained by the activation of NADPH oxidase and another mechanism which contributes to lipid-induced oxidative stress in β cells is the modulation of respiratory chain. β cells are also prone to the stress caused by low oxygen levels which leads to ROS production and other signs of oxidative stress. Hypoxia or low oxygen tension can lead to increased ROS generation, mostly through the involvement of complexes I and III of the mitochondrial electron transport chain. In the ER, ROS are generated as a product of protein folding events; therefore, the increased accumulation of dysregulated formation or breakage of disulfide bonds result in an excessive amount of ROS which causes oxidative stress. In addition, the presence of Superoxide Dismutase (SOD), causes the abnormal accumulation of H_2_O_2_ and other ROS (green box inset) which may damage the cells at multiple levels.

Indeed, excessive levels of ROS can directly damage the cells by oxidizing DNA, proteins and lipids, causing β cell dysfunction and death through different mechanisms, including changes in enzymatic activity, ion channel transport, receptor signal transduction, dysregulated gene expression, and apoptosis [32,33]. However, oxidative stress can also indirectly damage β cells by activating a variety of stress-sensitive intracellular signaling pathway mediators such as Nuclear Factor Kappa light-chain-enhancer of activated B cells (NF-κB), p38 Mitogen-Activated Protein Kinases (p38 MAPK), JNK/SAPK (c-Jun N-terminal kinase/Stress Activated Protein Kinases), hexosamine, and others. Nuclear targets for oxidative stress in the β cell are likely to include Pancreatic Duodenal Homeobox Factor 1 (PDX-1), which plays an important role in pancreas development and differentiation, as well as in maintaining normal β cell function [34]. This effect is mediated by the activation of the JNK pathway, causing the reduction of nuclear PDX-1 [35] and increased Nuclear Shuttling of Forkhead Box Protein O1 (FOXO1) [36]. Moreover, the rise of intracellular ROS can subsequently affect mitochondrial function, through depolarization of mitochondrial membrane potential (ΔΨ), thus reducing ATP production and insulin secretion [20,37]. Such results demonstrate that the main targets of ROS are mitochondria with consequent depolarization of mitochondrial membrane (ΔΨ) [38,39], ROS accumulation, and ATP depletion, suggesting that these mechanisms are tightly coupled among each other and related to the mitochondrial pathways of apoptosis [40] (Figure 1). 

Damages induced by ROS and/or the failure of antioxidant defenses, may cause several defects in insulin biogenesis and secretion in β cells, but also peripheral insulin-sensitive tissues dysfunction and damage, thus contributing to the onset of diabetes and its complications [16]. In fact, excessive ROS production caused by chronic hyperglycaemia also influences the development of secondary diabetic complications [41,42]. Potentially, in the onset and progression of late diabetic complications, free radicals have a major role due to their ability to damage macromolecules [43], leading to conditions that include coronary artery disease, neuropathy, nephropathy, retinopathy [42], and stroke [44].

## 3. miRNAs as Regulators of Oxidative Stress in Diabetes

As previously mentioned, miRNAs can be involved in the regulation of several key biological pathways and cellular functions demonstrating their critical role in insulin secretion, pancreatic development and function, and diabetic complications [45,46]. The alterations of specific miRNA levels can induce oxidative stress and damage and consequent development of different diseases. Therefore, it is very important to shed light on the role of miRNAs in β cell function and dysfunction, as well as in diabetes and its complications in oxidative stress conditions.

### 3.1. miRNAs, β Cell Function and Oxidative Stress

Pancreatic β cells are particularly susceptible to oxidative stress due to their deficiency in antioxidant defense mechanisms [47]. Several studies demonstrated a critical role for several miRNAs in β cell function and oxidative stress (reviewed in details in a manuscript of this special issue [48]).

As previously described, Reactive Oxygen Species (ROS) are considered important mediators of lipotoxicity and glucotoxicity in pancreatic β cells [49,50]. Studies have shown that human and murine pancreatic islets cultured at high glucose concentration results in miR-708 upregulation. Treatment with thapsigargin, a chemical inducer of Endoplasmic Reticulum (ER) stress, led to the upregulation of miR-708 inhuman islets as well as in ob/ob mouse islets; the treatment with chemical chaperone 4-phenylbutyrate, a molecule known to improve ER folding capacity [51], caused the inhibition of this miRNA, suggesting the involvement of ER stress in this response. Moreover, Neuronatin, previously shown to be involved in the modulation of the secretory function of β cells, has been identified as a potential target of miR-708. In fact, the expression of Neuronatin was reduced upon miR-708 hyperexpression and inversely correlated with miR-708 levels in human and murine islets exposed to different glucose concentrations. Of note, overexpression of Neuronatin restored the Glucose-Stimulated Insulin Secretion (GSIS) in murine and human islets cultured at low glucose levels. These results demonstrated that miR-708 could be used as potential target to restore β cell function under stress conditions [52,53].

As previously mentioned, high levels of Free Fatty Acids (FFAs) can induce oxidative stress in β cells. Indeed, prolonged exposure of cultured islet cells to palmitate caused an increase of miR-34a and miR-146a expression, leading to insulin-secretion dysfunction and β cell apoptosis. Additionally, inhibition of miR-34a and miR-146a expression protected islet cells from apoptosis but did not restore normal insulin secretion [9,54]. Another study confirmed the involvement of miRNAs in oxidative stress caused by FFAs. Indeed, in rat INS-1 (Insulinoma cell line 1) cells, the expression of miR-182-5p decreased after palmitate treatment and modulated the expression of Thrombospondin 1 (THBS‑1), a multifunctional glycoprotein associated to insulin resistance and β cell function [55]. Moreover, the incubation with THBS‑1, in INS-1 cells exposed to different concentration of palmitate, protected them against lipotoxic damage. 

The overexpression of THBS‑1 in INS-1 cells, abolished the negative effects of miR-182-5p on cell viability and apoptosis, suggesting the involvement of this miRNA in promoting β cell survival through the modulation of THBS-1 [56]. Importantly, another study demonstrated the protective effect of GLP-1 (Glucagon Like Peptide-1) against oxidative stress induced by FFA in rodent islets and INS-1 β cell line. In such a context, GLP-1 treatment was demonstrated to increase the expression of AMPK (Adenosine Monophosphate-Activated Protein Kinase) and its downstream dependent genes, including PPAR-α (Peroxisome Proliferator Activated Receptor Alpha), CPT-1 (Carnitine Palmitoyl Transferase-1), SREBP-1c (Sterol regulatory element-binding proteins 1-c), and SIRT1 (Sirtuin-1). It has been hypothesized that GLP-1 exerts its protective effects through the negative modulation of miR-33 and miR-370 expression, whose reduction improves β cell survival [57].

Of note, it has been reported that additional miRNAs may play a role in the induction of protection from metabolic stresses. Indeed, the deficiency of miR-200 in murine islets, led to a protection of β cells from oxidative stress through the repression of pro-apoptotic genes, indicating a critical role for this miRNA in the pathophysiology of diabetes [58]. Furthermore, it has been reported that the miR-200 family was hyperexpressed in pancreatic islets of diabetic mice, thus inducing β cell apoptosis. MiR-200 negatively modulates the expression of DNAJ heat shock protein family (Hsp40) Member C3 (Dnajc3) [an essential β cell chaperone known as Protein Kinase Inhibitor p58 (p58IPK)] and the caspase inhibitor X-Linked Inhibitor of Apoptosis Protein (Xiap), thus confirming its essential role in the regulation of β cell survival during oxidative stress conditions.

Recently, the effect of an antioxidant compound on β cells has been demonstrated to be mediated by miRNAs. Indeed, the antioxidant effects of treatment with Berberine (BBR), aisoquinoline alkaloid, have been analyzed in diabetic conditions. Importantly, in db/db diabetic mice, in streptozotocin induced diabetic mice, and in the mouse pancreatic β cell line NIT-1 (NOD/Lt mice) exposed to high glucose, the treatment with BBR led to a reduction of serum glucose, total cholesterol, and triglycerides. The levels of malondialdehyde, one of the oxygenated aldehyde chemicals, was reduced, while plasmatic levels of manganese-dependent Superoxide Dismutase 1 (SOD1), an effective endogenous antioxidant, were increased by BBR treatment in both animals and cells. BBR treatment is able to reduce oxidative stress injury by inhibiting miR-106b expression (associated with skeletal muscle insulin resistance) and consequent upregulation of Sirt1, whose function is to reduce the expression of pro-apoptotic molecules through Forkhead Box protein O1 (FoxO1) activation [59,60,61].

### 3.2. miRNAs, Oxidative Stress and Diabetic Complications

MiRNAs are differentially expressed in tissues/cells affected by chronic diabetic complications due to their modulation by several molecular mechanisms and transcription factors involved in oxidative and ER stress [8]. Several miRNAs have been analyzed in cellular and animal models of Diabetic Nephropathy (DN). Studies have demonstrated that miR-25 regulates the expression of NADPH oxidase and is involved in diabetic nephropathy. Indeed, the expression levels of miR-25 were significantly reduced in kidneys of diabetic rats as well as in high glucose-treated mesangial cells, thus causing the upregulation of NADPH oxidase 4 (NOX4), which promotes oxidative stress and is a key factor in the pathogenesis of DN. These results show that miR-25 is a pivotal factor involved in the regulation of NOX4 expression and function in DN [62]. In addition, NOX4 has also been shown to be a direct target of miR-146a in human endothelial cells [63]. MiR-146a causes an increased production of Extracellular Matrix (ECM) proteins, such as fibronectin in endothelial cells and in the retina of rats with glucose-induced diabetes [64]. This process is typical in the retina, kidney and heart in both type 1 and type 2 diabetes. In another study, it was shown that the expression of miR-205 is reduced in HK-2 (Human Kidney) renal tubular cell line upon hypoxia-reoxygenation-induced oxidative stress or ER stress. Furthermore, miR-205 negatively regulates Prolyl Hydroxylase 1 (PHD1/EGLN2) gene, which modulates the intracellular ROS level and ER stress state, thus playing a protective role against both oxidative and ER stresses [65].

The activation of Akt by Transforming Growth Factor-β1 (TGF-β) in diabetic kidneys plays an important role in the induction of fibrosis, hypertrophy, and cell survival in glomerular Mesangial Cells (MC). Indeed, it has been demonstrated that the activation of Akt by TGF-β leads to the upregulation of several miRNAs (miR-192, miR-216a, and miR-217), which negatively regulate Phosphatase and Tensin homologue (PTEN) by reducing the antioxidant gene Manganese Superoxide Dismutase (MnSOD) in mouse kidney MC [66]. Additionally, SOD1–2 are negatively regulated by miR-377 in human MC, and in combination with miR-217 caused a reduction of Heme Oxygenase-1 (HO-1) enzyme activity in Human Umbilical Vein Endothelial Cell line (HUVEC) [67]. Another study showed that miR-185 directly modulates the expression of the antioxidant enzyme Glutathione Peroxidases (GPx) which protects cells from oxidative damage. Indeed, it has been demonstrated that HUVEC cells exposed to oscillating glucose concentration (from 5 to 25 mmol/L), showed a reduced response of GPx in consequence of miR-185 overexpression [68].

Several authors also reported that miR-144 was reduced in left ventricles of streptozotocin induced diabetic mice and regulated high glucose-induced oxidative stress in cultured cardiomyocytes targeting Nuclear Factor-erythroid 2-Related Factor 2 (Nrf2), an important modulator of cellular response to oxidative stress. In fact, in diabetic mice, the inhibition of miR-144 enhanced Nrf2 expression in heart, reducing apoptosis, and improving cardiac function [69].

The expression of miR-214 was reported to be significantly reduced in the peripheral blood of rats with DN. On the other side, in human HK-2 renal proximal tubular epithelial cells, the upregulation of this miRNA caused a reduction of oxidative stress and ROS levels, but an increase of Uncoupling Protein 2 (UCP2), (p)-Akt, and p-Mammalian Target of Rapamycin (mTOR) protein expression levels [70]. Other authors suggested a strong relationship between miR-21 and acute hyperglycaemia. Indeed, in HUVEC cells exposed to constant high glucose and oscillating glucose (as a glucose variability model), miR-21 resulted in upregulation and promoted the suppression of ROS-homeostatic target genes such as KRIT1 (Krev/Rap1 Interaction Trapped-1), Nuclear Factor erythroid Related Factor 2 (NRF2), and MnSOD2 (Manganese-dependent Superoxide Dismutase2), which usually limits ROS damage. The post-trancriptional inhibition of these genes results, in turn, in mitochondrial dysfunction. On the other side, the inhibition of miR-21 in the same cell line improved the expression of SOD2 and KRIT1 leading to a reduction of ROS levels. These results suggest that the inhibition of miR-21 could represent a new therapeutic approach to limit the cellular oxidative injury due to glucose variability and diabetes [71]. Moreover, the same research group evaluated the expression of circulating miR-21 in a cohort (*n* = 109) of subjects, recruited in the DIAPASON (diabetes prediction and screening observation) study, and selected on the basis of American Diabetes Association (ADA) criteria for 2-h plasma glucose after a 75-g Oral Glucose Tolerance Test (OGTT). Recruited patients were classified into *n* = 39 NGT (Normal Glucose Tolerance), *n* = 43 IGT (Impaired Glucose Tolerance) and n = 27 T2D (Type 2 Diabetes), newly diagnosed and free from drug treatment. They observed that miR-21 was upregulated in IGT and in T2D with respect to NGT subjects, and was positively correlated with glycaemic parameters. Furthermore, in IGT and T2D subjects there was a significant overproduction of ROS, detected by Electron Paramagnetic Resonance (EPR), an accumulation of lipid peroxidation marker 4-HNE (4-Hydroxynonemal) and defective SOD2 antioxidant response. These results suggest that miR-21 is an early predictor of ROS damage prior to the onset of diabetes and could represent a promising biomarker and a therapeutic target as well [72].

As previously described, H9c2 cells (Embryonic Cardiac myoblast cell line), exposed to high glucose, show a reduction of viability, an increase in the production of ROS and a decrease of SOD levels. Of note, it has been demonstrated that in streptozotocin-induced diabetic mice and in high-glucose-treated H9c2 cells, miR-22 overexpression restored cells viability, reduced the injury caused by oxidative stress and reversed cardiac dysfunctions. Furthermore, miR-22 overexpression reduced oxidative stress injury in diabetic cardiomyopathy by enhancing SIRT1 expression both in vivo and in vitro [73].

A recent study demonstrated that miR-92a is upregulated in the aortic endothelium of db/db mice and in renal arteries of diabetic patients, due to the exposure of Endothelial Cells (ECs) to Advanced Glycation End-products(AGEs) and oxidized low-density lipoprotein [74]. Interestingly, the inhibition of miR-92a in db/db mice protects endothelial cells function by normalizing ROS generation and by improving Heme-Oxygenase-1 (HO-1) expression. Thus, miR-92a-HO-1 pathway may represent a valid mechanism against diabetic vasculopathies induced by oxidative stress.

Another study demonstrated that miR-15a can induce oxidative stress in retinal Müller glial Cells (rMC-1) through the modulation of Akt3. miR-15a is overexpressed in the plasma of patients with Diabetic Retinopathy (DR) and is transferred to retinal cells through exosomes secreted by β cells, where it plays a pivotal role in insulin production and secretion. This study highlights an important mechanism of cell-cell communication in which circulating miR-15a released by β cells, may influence the function of another cell type being involved in disease progression [75].

The role of miR-365 in retinal cells function and retinopathies has also been explored. The expression of miR-365 was upregulated in the Muller cell line treated with glyoxal and in the retina of rats affected by DR. Moreover, Timp3 (metalloproteinase inhibitor 3), (previously shown to be involved in the protection from oxidative stress mechanisms) was negatively regulated by miR-365; indeed, the inhibition of miR-365 led to an increase of Timp3, thus inducing a relief for Muller cell gliosis and oxidative stress. In the light of these findings, the authors suggested that miR-365 and Timp3 may represent potential therapeutic targets for the treatment of DR and other oxidative stress-related retinopathies [76]. Collectively, such results confirmed the involvement of miR-365 in the pathogenesis of DR through exacerbation of oxidative stress.

Additional specific miRNAs have been suggested as mediators of induced-protection against oxidative stress in diabetes. Indeed, in a recent study, ob/ob mice were treated with Unacylated Ghrelin (UnAG) in order to protect skeletal muscle and endothelial cells from oxidative stress. The authors demonstrated that treatment with UnAG induced the upregulation of miR-126, thereby causing an increase of SIRT1 expression and a reduction of Histone-3 Lysine-56 (H3K56) deacetylation. These data indicated that UnAg protected endothelial cells from ROS imbalance by reducing cell senescence through the modulation of miR-126 expression in ob/ob mice [77]. Another miRNA reported to be involved in diabetes-related oxidative stress is miR-34a, a p53-regulated miRNA, hyperexpressed in aortic endothelium of db/db and streptozotocin-induced diabetic mice. The endothelium-specific knock out of miR-34a prevents the downregulation of aortic SIRT1 expression, thus avoiding endothelium-dependent aortic vasorelaxation induced by diabetes. In addition, in endothelial cells the increase of H_2_O_2_ and the induction of endothelial miR-34a, due to high glucose or palmitate, is repressed by knockdown of p66shc, a master promoter of oxidative stress. These results indicated that upon chronic hyperglycaemia and high exposure to free fatty acids, p66shc recruitment leads to the upregulation of endothelial miR-34a which causes endothelial dysfunction by targeting SIRT1 [78] (Table 1).

## 4. MiRNAs as Therapeutic Targets: Strategies and Perspectives

miRNAs have been largely associated with several diseases and pathologic alterations, including oxidative stress both in β cell dysfunction and diabetic complications. Thanks to their ability to target a large number of genes, miRNAs represent upstream regulators of many cellular physiological and pathological processes [79]; hence, the direct targeting of a single miRNA (or a set of miRNAs) could potentially lead to the modulation of an entire biological process. Several therapeutic strategies aimed at modulating miRNAs expression in specific cell types or tissues have been largely investigated and represent one of the most intriguing research area.

### 4.1. miRNA Mimicking or Inhibition

Specific miRNA(s) hyperexpression or hypoexpression have been observed in multiple cells/tissues in several diseases [80,81,82], leading to downregulation or upregulation of their target genes respectively, thus causing alterations of entire cellular processes. Therefore, potential therapeutic strategies aimed at restoring physiological miRNA expression levels can be based both on miRNA mimics or miRNA inhibitors, depending on the type of alteration observed (hypoexpression or hyperexpression). In order to restore the expression of a downregulated miRNA, a mimicking strategy has been explored. MiRNA mimics are double-stranded non-natural small RNA molecules whose function is to mimic and replace natural-borne miRNAs [83]. Importantly, miRNA mimics can be fully designed and chemical modifications adapted based on experimental requirements [84,85].

As for miRNA inhibition strategies, two different methods have been explored with the aim to inhibit the expression of miRNAs: (i) miRNA sponges; (ii) antisense oligonucleotides.

MiRNA sponges are tandem-repeated miRNA target sequences located after a reporter gene, acting as a miRNA decoy in order to prevent its binding to a target mRNA [86]. Although miRNA sponges can be naturally found within long non-coding RNA sequences in plants [87] and animals [88], they can also be synthetically produced as plasmid or viral vectors containing tandem arrayed miRNA binding sites separated each other by small nucleotide sequence spacers and inserted into a 3′UTR (Untranslated Region) portion of a reporter gene driven by an RNA polymerase II promoter [89]. Another natural miRNA sponge is represented by endogenous circular RNA produced by back-splicing (an alternative RNA splicing) [90].

Antisense oligonucleotides, also known as antagomiRs, are RNA sequences that are highly complementary to the target miRNA; indeed, they bind to the miRNA guide strand thus inhibiting its function, or degrade it via recruitment of RNase H [91].

However, both miRNA mimics and inhibitors are naturally subjected to degradation by nucleases [92]; chemical modifications are needed in order to be used for in vivo therapies. Several modifications have been described to counteract nucleases degradation, such as 2′-O-methyl, cholesterol tail and phosphorothioate [93,94].

Of note, a novel type of oligonucleotide (highly stable and highly resistant to degradation) is represented by Locked Nucleic Acids (LNA) based oligos. LNA are bicyclic nucleic acids that bind the 2′O to the 4′C, locking sugar into a 3′ endo conformation [95], thus significantly improving a sequence’s stability and specificity. As a matter of fact, the first miRNA-based drug was developed using LNA containing phosphorothioate modifications and specifically targets miR-122 by inhibiting its expression. MiR-122 is essential for the survival of Hepatitis C Virus (HCV) in the liver [96,97]. Indeed, the interaction of miR-122 with the HCV Internal Ribosome Entry Site (IRES) is required for the HCV replication cycle and involves the recruitment of Argonaute proteins to induce HCV polyprotein translation through enhancement of IRES function [98] and stabilization of the viral RNA [99]. Since the HCV replication cycle is dependent on this mechanism, many research groups have started to investigate the potential use of miR-122 antagonists to inhibit HCV replication. The recently reported phase IIa clinical trial in humans, supported by Santaris Pharma, confirmed that Miravirsen (the commercial name of the LNA anti-miR-122) inhibits the human HCV replication cycle; indeed, its effectiveness was observed in 36 patients infected with HCV genotype 1 [100]. These data demonstrate that the targeting of specific miRNAs may represent an efficient therapeutic strategy to treat several diseases.

### 4.2. Delivery Systems for miRNA-Based Drugs

**Figure 2:**
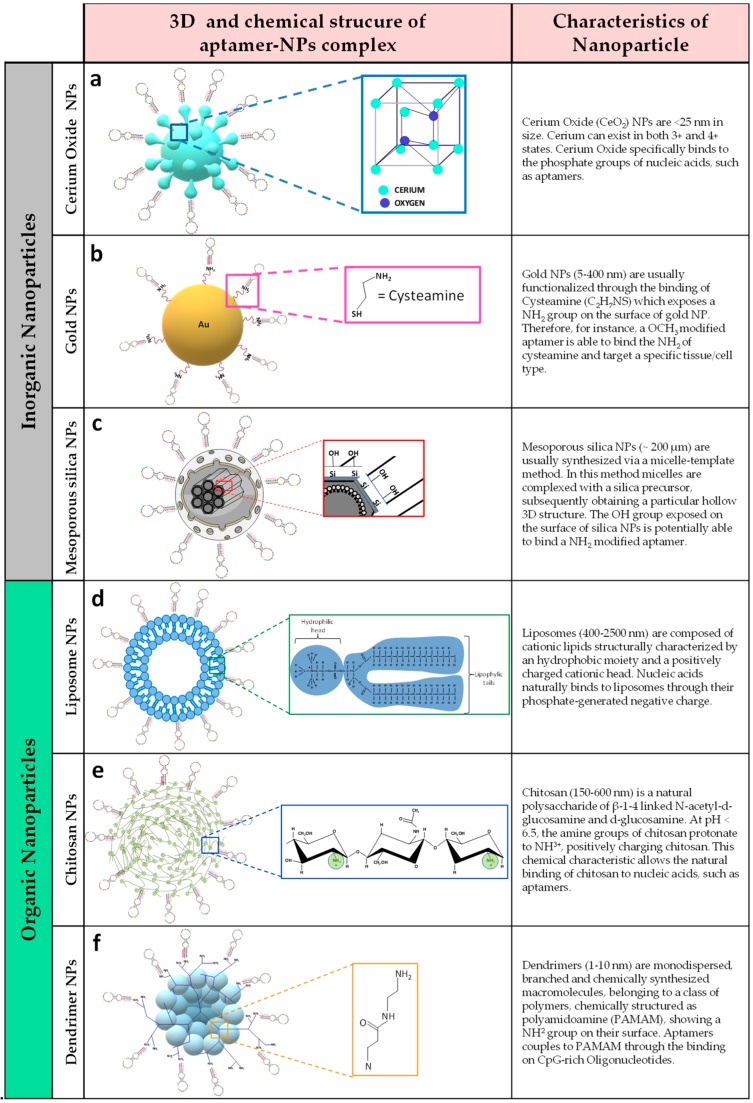
3D structure and details of chemical composition of inorganic and organic Nanoparticles (NPs) conjugated to aptamers/nucleic acids. (**a**) Cerium oxide NP 3D structure is due to the aggregation of several cerium oxide molecules. (**b**) Gold NPs are modified with Cysteamine (Cys); this modification on gold NP is mainly used to stabilize its binding to the target drug. (**c**) The hollow 3D structure of a mesoporous silica NP is represented as well; briefly, this structure is composed by a combination of micelles complexed with a silica precursor (SiOH) on the surface of the NP (magnified in the red box). (**d**) 3D structure of a liposome NP and detailed chemical structure of a single phospholipid composing the membrane of liposome (green box). (**e**) Chitosan-composed NP is displayed, alongside with the chemical structure of a positively charged chitosan macromolecule on the NH_3_ group (green circle). (**f**) Branched chemical structure and 3D composition of a Polyamidoamine (PAMAM) dendrimer is shown; in the orange box, the typical chemical structure of a PAMAM dendrimer branch is represented.

One of the most critical aspects of targeted in vivo miRNA modulation (both inhibition and mimicking) is represented by the lack of a safe and reliable strategy to specifically target organs and tissues/cells. Different inorganic and organic nanoparticles as well as the novel developed aptamer-based technology, are currently being investigated as vehicles to deliver miRNA modulation complexes, in order to specifically target cells and tissues in an effective, non-cytotoxic manner (Figure 2).

#### 4.2.1. Inorganic Nanoparticles

Inorganic Nanoparticles (NPs) have striking electrical and optical properties, as well as high biocompatibility [101]. Among the most cited and characterized inorganic NPs adopted for miRNA delivery, gold NPs and mesoporous silica NPs are noteworthy [102]. However, cerium oxide NPs can also be a valid alternative, due to their intrinsic antioxidant properties [103].

The inorganic nanomaterial mainly used to deliver miRNA drugs is based on gold NPs due to their stability, surface modification ability, and high biocompatibility [104,105,106]. MiRNAs bound to gold nanoparticles have been mainly developed by using cysteamine-functionalized gold nanoparticles (AuNPs). Cysteamine (Cys), a sulfur-containing biomolecule, is frequently used as a stabilizer for AuNPs [107] as well as a linking agent on the surface of AuNPs [108]. Due to the strong Au-S bonds, Cysteamine can be easily linked to AuNPs while the −NH_2_ group can exposed on the surface of Cysteamine-capped gold nanoparticles (Cys-AuNPs), thus being a link for several biomolecules [109].

Another promising delivery system is based on mesoporous silica material; indeed, mesoporous silica nanoparticles have been previously described as novel and ideal nanomaterial for the delivery of RNA-based drugs [110,111,112]. They are synthesized by the reaction of tetraethyl orthosilicate with a template made of micellar rods. The result is a 3D structure composed of nano-sized spheres or rods filled with a regular arrangement of pores [113]. The first silica nanoparticle-miRNA-conjugated drug was linked to miR-34a, which was administrated to neuroblastoma tumor-bearing mice. As a matter of fact, it was demonstrated that the administration of silica-NPs linked to miR-34a, elicited tumor suppressive and pro-apoptotic activity of cancer cells [114].

Cerium oxide Nanoparticles (CeNPs) have been shown to represent a valid carrier for miRNA delivery, particularly asit has been demonstrated to have antioxidant properties [103]. The bio-relevant activities of CeNPs render them promising pharmacological agents [115], drug delivery carriers [116], and bioscaffolding molecules [117]. Importantly, research efforts to identify and characterize the biomedical applications of CeNPs has been focused on the investigation of their use to treat diseases characterized by high levels of Reactive Oxygen Species (ROS) [118]. Cerium is the most abundant among the seventeen rare earth metals belonging to the lanthanide series, and is present in two oxidation states: +3 and +4. Cerium oxide, also called noceria, is considered to be a lanthanide metal oxide and is used as an ultraviolet absorber, catalyst andgas sensor. Noceria can be synthesized through chemical (precipitation, such asco-precipitation and chemical precipitation, microwave, sonochemical, hydrothermal, etc.) or natural methods (from *Gloriosa superba* leaf extracts or *Curvularia lunata* culture filtrate) [119]. Of note, Zgheib and colleagues demonstrated that miR-146a mimics (hypoexpressed in diabetic wounds and involved in inflammatory processes in diabetes) conjugated to cerium oxide nanoparticles, were able to reduce ROS overproduction and oxidative stress occurring in epithelial cells of diabetic wounds of db/db mice, with a consequent healing enhancement and improved biomechanical properties of the healed skin. In the same study, these results were also confirmed in a porcine model of streptozotocin induced diabetes. In this model, diabetic wounds treated with miR-146a conjugated to cerium oxide NPs (miR-146a-CeO-NP) were significantly smaller in size respect to the wounds treated with PBS (Phosphate Buffer Saline). Moreover, diabetic wounds of the same pigs treated with miR-146-CeO-NP also showed a decreased frequency of CD45^+^ immune cells, indicating a reduction of inflammatory processes [120].

Among others inorganic NPs, Graphene Oxide (GO) has attracted much interest. Graphene is a two-dimensional nanomaterial largely studied for its excellent physical, chemical, and mechanical properties [121], rendering it an optimal delivery drug carrier [122]. In addition to GO, other inorganic metallic nanomaterials such as iron oxide and silver oxide have been also investigated as potential carriers for drug delivery strategies [123]. However, despite their optimal properties, these types of nanomaterials induce important alterations in ROS production and in cellular responses to oxidative stress and therefore should be excluded from the plethora of therapeutic delivery strategies to treat oxidative stress [124].

#### 4.2.2. Organic Nanoparticles (NPs)

miRNAs can easily be transferred to cells and tissues using liposomes [102,125]. Liposomes are composed of cationic lipids structurally characterized by an hydrophobic moiety and a positively charged cationic head; nucleic acids naturally binds to liposomes through their phosphate-generated negative charge [126]. In addition, liposomes have low toxicity and a consequent low risk of immunological rejection, which represents one of the most important issue in drug-delivery strategies [127]. Pramanik and colleagues developed lipid-based NPs to deliver miR-34a mimics with the aim to limit pancreatic cancer cell growth in mice. Interestingly, the administration of miR-34a-lipid-NP was able to reduce tumor growth [128]. Of note, the liposome-miR-34a drug, MRX34, is now under clinical phase I for the treatment of solid tumors [129]. Importantly, miR-34a is able to target and regulate the expression of Sirtuin-1 (SIRT1) [130], whose upregulation is critical to inhibit oxidative damage to vascular endothelial cells [131]. Of note, the expression of miR-34a has been found upregulated in subjects with diabetes-induced chronic heart failure [132]; consequently, a nano-drug aimed at inhibiting the expression of miR-34a could represent a novel and effective approach to treat oxidative stress in T2D vascular complications [133].

An additional strategy is represented by polymer-mediated delivery of miRNAs. Dendrimers have been reported as an optimal nanomaterial to generate miRNA-based drugs. Dendrimers are monodispersed, branched, and chemically synthesized macromolecules, belonging to a class of polymers, mainly used to deliver oligonucleotides [134]. Interestingly, Wang and colleagues encapsulated miR-34a mimic into S6 aptamer-conjugated-dendrimer in order to deliver a lung cancer-targeted gene nanoparticle (PAM-Ap/pMiR-34a NPs). As expected, aptamer conjugation has significantly improved cellular uptake as well as gene transfection efficiency of PAM-Ap/pMiR-34a NPs in cultured Non-Small-Cell Lung Cancer (NSCLC) cells. Furthermore, PAM-Ap/pMiR-34a NPs was efficient to modulate the expression of B Cell Lymphoma 2 (BCL-2) and p53genes in vitro, inhibiting cell growth, migration, and invasion, and inducing apoptosis of lung cancer cells, compared with non-targeted NPs [135].

Chitosan is a natural polysaccharide of β-1-4 linked *N*-acetyl-d-glucosamine and d-glucosamine, showing natural abundance, low toxicity [136], biodegradability, and mucoadhesivity; such properties make this material ideal to deliver miRNA-based complexes [137]. Currently, chitosan-conjugated miRNA drugs are mainly developed for cancer therapy, such as miR-145/chitosan complex (breast cancer) [136] or miR-34a/chitosan complex (bone metastases caused by prostate cancer) [138]. Overall, these data show that the function of miR-34a has been largely studied, and that its modulation has been delivered through different NP formulations. Indeed, the administration of miR-34a mimics conjugated with several NPs has been demonstrated to inhibit tumor growth and progression. However, since miR-34a has been found upregulated in oxidative stress conditions in diabetes and diabetic complications, the inhibition of its expression could represent a potential strategy to treat oxidative stress damage in diabetes.

#### 4.2.3. Aptamers

One of the most potentially promising strategies to develop drugs aimed at modulation of miRNAs with high specificity for cells and tissues is represented by aptamers. Aptamers are short single-stranded (ss) DNA or RNA molecules selected for the binding to a specific target [139]. Aptamers can fold into different secondary structures, such as stem loop, bugle, pseudoknot, G-quadruplex, and kissing hairpin [140], resulting in the formation of a unique three-dimensional (3D) structure able to specifically recognize their targets [141]. The 3D interactions are pivotal for aptamer binding affinity, and specificity, and drives the formation of aptamer-target complexes [142]. Aptamers are generated through the SELEX (Systematic Evolution of Ligands by Exponential Enrichment) methodology, which consists of a series of selection cycles aimed at increasing aptamers affinity to a specific target [143]. Upon several rounds, aptamers libraries are strictly selected based on experimental conditions (pH, binding temperature, binding time, etc.) and on target properties (charge, hydrophilicity/hydrophobicity, etc). These variables contribute to the specific selection which affects the affinity and function of the enriched aptamers [144]. 

Different procedures are adopted to generate DNA or RNA aptamers. DNA aptamers are generated through incubation of the target with the library, which is then amplified with Polymerase Chain Reaction (PCR), thus resulting in a dsDNA (double stranded DNA) library subsequently subjected to strand separation to produce a new ssDNA library for the next selection cycle. On the other side, RNA aptamersare subjected to reverse transcription into dsDNA to enable subsequent RNA transcription [145]. DNA aptamers are inherently more stable and the related manufacturing costs are lower than RNA aptamers [146]. RNA aptamers have diverse 3D conformations, and stronger intra-strand RNA-RNA interactions, which increases binding affinity and specificity [147]. However, unmodified aptamers are susceptible to nuclease-mediated degradation which causes short half-lives in vivo [148]. For this reason, aptamers with a putative clinical application should be chemically modified. 

Generally, aptamers are modified by replacing the 2′ position with different chemical groups, such as a fluoro- (F), amino- (NH_2_), or O-methyl (OCH_3_) group, and by capping the 3′ end with inverted thymidine in order to increase nuclease resistance and enhancing binding affinity. After these modifications, “remodeled” nucleotides can be incorporated into aptamers through two different strategies: in-SELEX and post-SELEX. In the in-SELEX strategy, modified aptamers are directly isolated from a DNA or RNA library containing modified nucleotides compatible with DNA or RNA polymerases [149]. In post-SELEX strategies, modified aptamers are inserted in pre-selected aptamers during solid-phase chemical synthesis. However, since the affinity and the specificity as well as the function of an aptamer is sensitive to its structure, post-SELEX modification may affect the inherent properties and folding structures of the original aptamers, thereby compromising the binding affinity [150]. Therefore, specific modifications should be precisely chosen depending on aptamer molecule clinical application, taking into consideration the potential alteration of therapeutic efficacy or side-effects. Importantly, aptamers have also been identified as valid vehicles for miRNA-drugs delivery [151]. Indeed, different studies describe the efficacy of miRNA mimics or inhibitors conjugated to aptamers. For example, Li and colleagues demonstrated that miR-188 is upregulated in Bone Marrow Mesenchymal Stem Cells (BMSCs), and that its overexpression leads to bone loss and bone marrow fat accumulation. In order to inhibit this age-related condition, the authors developed an aptamer conjugated to antagomiR-188, that specifically targets BMSCs (respect to macrophages/monocytes and preosteoclasts). The results show that miR-188 inhibition through the aptamer delivery system is able to restore bone formation and to block fat accumulation [152]. On the other side, Iaboni and colleagues designed an aptamer-miRNA chimera system that consists of GL21.T, an RNA aptamer capable of recognizing the tyrosine kinase receptor Axl and miR-212. This strategy allows to the chimeric aptamer to specifically target NSCLC cells. In addition, the authors demonstrated that the conjugation of miR-212 to GL21.T efficiently inhibits the anti-apoptotic protein PED/PEA-15 (15 kDa Phosphoprotein Enriched in Astrocytes), involved in the tumor resistance to chemotherapy treatment. Moreover, this chimera system is able to increase the caspase activation and reduce the viability of tumor cells, in combination with Tumor Necrosis Factor(TNF)-related apoptosis-inducing ligand (TRAIL) therapy [153].

### 4.3. Clinical Advancements of miRNA-Based Drugs

Among those miRNAs previously described to have a role in the regulation of molecular mechanisms of oxidative stress, some of them have been suggested as therapeutic targets. For example, miR-21-conjugated NPs has been assessed in glioblastoma treatment [154,155]. Specifically, Seo and colleagues demonstrated that both RNA-based anti-miR-21 conjugated with a cationic Poly(Amine-Co-Ester) (PACE) and Peptide Nucleic Acid (PNA) anti-miR-21 conjugated with a copolymer of Poly(Lactic Acid) and Hyperbranched Polyglycerol (PLA-HPG), provided an efficient intracellular delivery system to inhibit miR-21 expression, leading to PTEN upregulation and apoptosis of human GBM (Glyoblastoma Multiforme) cancer cells. MiR-21 has also been demonstrated to be hyperexpressed in HUVEC (Human Umbelical Vein Endothelial Cells) cells uponconstant or oscillating high glucose exposure, and its upregulation is able to inhibit expression and function of several specific target genes such as KRIT1(Krev/Rap1 Interaction Trapped-1), NRF2 (Nuclear Factor erythroid Related Factor 2), and SOD2 (Superoxide Dismutase 2) (which have anti-oxidant functions), thus generating mitochondrial dysfunction. Circulating miR-21 was also found upregulated in T1D, T2D, and in IGT patients, in which increased ROS production has been observed [72]. Moreover, as antagomiR-21 conveyed with different NPs resulted in efficient uptake by target tissue, a miR-21 nano-based drug could also be promising for diabetes therapies targeting oxidative stress.

Recently, a GalNac (*N*-Acetylgalactosamine)-conjugated oligonucleotide linked to miR-103 and -107 inhibitors (RG-125,AZD4076) was developed for the treatment of non-alcoholic steato hepatitis (NASH) in patients with T2D. Currently, a clinical phase I trial with RG-125, as therapy for patients with T2D and liver disease is currently ongoing (ClinicalTrials.gov:NCT02826525). However, to date, there is no evidence that these drugs are efficient to inhibit oxidative damages occurring in diabetes and its complications.

Another important miRNA, whose role in the protection against oxidative stress has been largely assessed, is the angiomiR miR-126. Importantly, Deacetylated poly-*N*-Acetyl glucosamine (DEAC-pGlcNAc) polymers miR-126-NPs have been developed [156] for the treatment of sepsis; this complex resulted in an appropriate size for cellular uptake, as well as ideal to avoid degradation by RNAses. Of note, miR-126 regulates SIRT1 (Sirtuin 1) and is able to protect endothelial cells from ROS-induced senescence [77], and has been demonstrated to be involved in angiogenic signaling and vascular integrity [157]. Collectively, such results suggest that miR-126 may represent a potential therapeutic target in order to reduce oxidative stress and vascular damage during diabetes.

Finally, several studies suggest that miR-146a may represent an additional therapeutic target in diabetes, given its important role in inflammation and oxidative stress both in T1D and T2D. Indeed, miR-146a-polymer-based-NPs beneficial effects have been demonstrated in diabetes-induced peripheral neuropathy [158]. Of note, miR-146a has been shown to directly target NOX4 (NADPH Oxidase 4) [63], causing an increased production of Extracellular Matrix (ECM) proteins in endothelial cells and in the retina of rats with glucose-induced diabetes [64], thus potentially representing a candidate target for the treatment of diabetic neuropathy.

## 5. Concluding Remarks

Chronic hyperglycaemia is the primary cause of severe tissue damages, induced by several important mechanisms involved or activated in consequence of oxidative stress [159,160]. Among the epigenetic factors that regulate oxidative stress mechanisms, miRNAs have been demonstrated to play an essential role [161]. In fact, a large number of miRNAs have been demonstrated to regulate the expression of several factors causing or preventing oxidative stress in T1D, T2D, or diabetic complications.

Several innovative drugs based on miRNA modulation have been developed for the treatment of different diseases. Among these, aptamers show the essential ability to target a particular miRNA in a specific cell type, avoiding the onset of side/off-target effects in other cells/tissues.

Although several advancements have been made in the design of novel drugs, the development of innovative strategies and high efficient delivery systems aimed at modulating miRNA expression is still highly required in order to treat diabetes dysfunctions and its chronic complications.

## Figures and Tables

**Table 1:** microRNAs involved in oxidative stress mechanisms occurring in diabetes and its complications. The table reports the microRNAs up (↑) or downregulated (↓) in diabetes and its complications and involved (directly or indirectly) in oxidative stress processes. Tissue/cells involved, experimentally validated target genes, their function in oxidative stress, and disease details are reported as well. T2D, Type 2 Diabetes; DN, Diabetic Nephropathy; DV, Diabetic Vasculopathy; DC, Diabetic Cardiopathy; DR, Diabetic Retinopathy; IGT, Impaired Glucose Tolerance.

MicroRNA	Cell/Tissue	Target Gene	Target GeneFunction	Disease or Dysfunction	Ref.
miR-708 ↑	- Mouse pancreatic islets (ob/ob mice) - Min6 β-cell line; Ins1 β-cell line	Neuronatin↓	Overexpression of Neuronatin restores β cell function under ER stress	T2D	[51,52,53]
miR-34a ↑	- Min6 β-cell line - Islets from C57/Bl6 KsJ db/db - Rat pancreatic islets			T2D	[9,54]
miR-146 ↑	- Min6 β-cell line - Islets from C57/Bl6 KsJ db/db - Rat pancreatic islets			T2D	[9,54]
miR-182-5p ↓	- Visceral and subcutaneous adipose tissue from human donors - Ins1 β-cell line	THBS-1 ↑	Upregulation of THBS-1 protects β cells from lipotoxic damage	T2D	[55,56]
miR-370 ↓ miR-33 ↓	- Ins1 β-cell line - C57BL/6 mice HFD			T2D	[57]
miR-200 family ↑	- db/db mice - Min6 β-cell line	p58IPK/XIAP ↓	Physiologic expression of p58IPK/XIAP protects the β cells from oxidative stress	T2D	[58]
miR-106-b ↓	- db/db mice - NIT-1 β-cell line	SIRT-1 ↑	SIRT-1 upregulation leads the reduction of pro-apoptotic molecules expression through FoxO1 activation	T2D	[59,60,61]
miR-25 ↓	- Diabetic rat streptozotocin-induced	NOX-4 ↑	Upregulation of NOX-4 promotes oxidative stress	DN	[62]
miR-146a ↓	- Human Umbilical Vein Endothelial Cells (HUVECs)	NOX-4 ↑	Upregulation of NOX-4 promotes oxidative stress	DV	[63,64]
miR-205 ↓	- HK-2 cell line	PHD1/EGLN2 ↑	Upregulation of PHD1/EGLN2 modulates intracellular ROS level and ER stress state	DN	[65]
miR-192miR-216a miR-217 ↑	- C57/Bl6 db/db mice - Mouse mesangial cells	PTEN ↓	Downregulation of PTEN leads the reduction of MnSOD and its antioxidant activity	DN	[66]
miR-377 ↑	- Human MC	SOD-1/SOD-2 ↓	Physiologic expression of SOD-1-2 protects cells from ROS	DN	[67]
miR-217miR-377 ↑	- HUVEC cell line - HEK-293 cell line	HO-1 ↓	Downregulation of HO-1 leads to impaired metabolization of excessive heme generate during hemolysis	DN	[67]
miR-185 ↑	- HUVEC cell line	GPx↓	Physiologic expression of GPx protects cell from oxidative damage	DC	[68]
miR-144 ↓	- C57BL/6 mice diabetic STZ induced	Nrf2 ↓	Upregulation of Nrf2 reduces apoptosis and improving cardiac function	DC	[69]
miR-214 ↓	- Male Sprague Dawley rats diabetic STZ induced - Human HK-2 cell line	UCP2 ↑	UCP2 inhibition attenuates the effects of miR-214 upregulation on oxidative stress	DN	[70]
miR-21 ↑	- HUVEC cell line	KRIT1/NRF2/SOD2↓	Physiologic expression of KRIT1/NRF2/SOD2 limits ROS damage	DC	[71]
miR-21 ↑	- Human plasma			IGT/T2D	[72]
miR-22 ↑	- C57BL/6 mice diabetic STZ induced - C57BL/6 mice HFD - Rat H9c2 cell line	SIRT-1 ↑	SIRT-1 upregulation protects from oxidative stress	DC	[73]
miR-92a ↓	- C57BL/6 db/db mice - HUVEC	HO-1 ↑	HO-1 upregulation normalizes ROS generation	DV	[74]
miR-15a ↑	- Human plasma			DR	[75]
miR-365 ↑	- Rat Muller cell line - Sprague Dawley rats	TIMP-3 ↓	Overexpression of Timp-3 improves Muller cell gliosis and retinal oxidative stress	DR	[76]
miR-126 ↑	- C57BL/6 ob/ob mice - Endothelial cells	SIRT-1 ↑/H3K56 deacetylation↓	Upregulation of SIRT-1 and reduction of H3K56 deacetylation protects cells from ROS	T2D	[77]
miR-34a ↑	- db/db mice	SIRT-1 ↓	SIRT-1 upregulation protects from oxidative stress	T2D	[78]
